# Isolation to purification: An integrated study on lysine production by *Bacillus megaterium* NTJT4 from medicinal plant rhizosphere

**DOI:** 10.1371/journal.pone.0361763

**Published:** 2026-10-30

**Authors:** Taslimul Jannat, Md. Rezaul Karim, Md. Rakibul Hasan, Debabrata Karmakar, Nishat Tasnim, Nourin Tarannum

**Affiliations:** Institute of Technology Transfer and Innovation (ITTI), Bangladesh Council of Scientific and Industrial Research (BCSIR), Dhaka, Bangladesh; Benemérita Universidad Autónoma de Puebla: Benemerita Universidad Autonoma de Puebla, MEXICO

## Abstract

L-lysine stands out as a commercially important amino acid vital for maintaining balanced nutrition, immune function and healthy development in humans and animals. As global demand for lysine continues to rise each year, large-scale production is required to keep pace with this growing need. In the present study, L-lysine production was accomplished through a fermentation process utilizing isolated bacterial strains from soil. After conducting primary screening, *Bacillus megaterium* NTJT4 was identified as the highest lysine producer. Various fermentation parameters including fermentation time, pH, temperature, different carbon and nitrogen sources, glucose levels, ammonium sulfate concentration, growth stimulators, and vitamin concentrations were evaluated to optimize lysine production. The study found that the optimal conditions for lysine production were 72 hours, pH 7.0, and 37 °C. The effect of different carbon and nitrogen sources showed that 8% glucose and 1% ammonium sulfate resulted in increased lysine production (4.86 ± 0.20 mg/mL and 3.95 ± 0.14 mg/mL respectively). Media with combination of peptone and yeast extract as growth stimulators showed increased lysine yield (4.86 ± 0.31 mg/mL) and addition of 100 µg/mL thiamine-HCL as a source of vitamin resulted in increased lysine yield (5.37 ± 0.16 mg/mL). Amberlite IR‑120–based ion‑exchange chromatography achieved 100.0% purity and 58.0% recovery of lysine. The purified lysine concentration reached 13.44 mg/mL with a purification fold of 3.85. These results indicate that the newly isolated *Bacillus megaterium* NTJT4 possesses considerable potential for diverse biotechnological and industrial applications, particularly in microbial amino acid production.

## Introduction

Proteins are the most essential macromolecules for both human and animal functions, and amino acids constitute their fundamental structure. The appropriate growth, development, and maintenance of health depend on essential amino acids. L-lysine is one of the nine L-amino acids that are necessary for human and animal nutrition out of the 20 L-amino acids present in the majority of living things and so the demand for it has been rising rapidly in recent years [1]. With several hundred thousand tons (about 800,000 tons) produced annually, it is the second amino acid manufactured on a major industrial scale [2, 3]. L-lysine is typically found to be the most inadequate ingredient in animal and human diets. Since cattle, poultry, and other animals cannot synthesis L-lysine and is present in animal feed in trace amounts, L-lysine will be supplied to the animal’s diet to meet feed needs [4]. Since L-lysine is thought to be the primary limiting amino acid found in every known cereal morsel, boosting the protein content of cereal-based diets requires more skill, particularly in developing nations where cereal crops are heavily relied upon. L-lysine supplementation enhances the protein quality of wheat-based meals, which promotes better tissue synthesis and growth. Additionally, it has pharmacological uses in both the infusion of amino acids and the creation of diets with a balanced amino acid composition. According to reports, adding 0.4% L-lysine (HCl salt) and 0.15% L-threonine to wheat flour produces material that has the same nutritional content as milk; nevertheless, animal feed can be strengthened by combining 0.1% lysine and DL-methionine [5]. In the human body, it is also utilized for the creation of hormones, enzymes, and antibodies as well as antihypertensive and analgesic neutralizers [6].

Protein hydrolysis, chemical and microbiological synthesis are the processes used to produce amino acids. The direct-fermentation approach has been used to manufacture the majority of L-lysine from natural carbon sources [7]. However, chemical methods of synthesizing L-lysine cause significant environmental contamination, necessitate numerous steps and tools, are expensive to produce, and include hazardous materials including hydrogen cyanide, propylene, and methyl mercaptan [8]. Because it uses low temperature, low pressure, and inexpensive carbon sources to produce a biological form of lysine, a low-cost, environmentally friendly production of pure lysine using microorganisms based on natural renewable resources is therefore becoming more appealing and necessary [9]. The following microorganisms have been shown to produce L-lysine: *Bacillus* *megaterium* [10], *Brevibacterium linens*, *Streptomyces albulus IFO* [11], *Brevibacterium flavum* [12], *M. methylophilis* [13], *B. subtilis,* and *Bacillus laterosporus* [14]. A broad spectrum of nitrogen sources is applicable, ranging from inorganic and organic salts, including ammonium salts, to organic compounds like urea. Additionally, various proteolytic materials—such as peptone, casein hydrolysate, yeast extract, corn steep liquor, and soybean protein hydrolysate—as well as other plant and animal tissue extracts, may serve as effective nitrogen contributors [5]. Lysine is typically obtained in the form of a salt, most often lysine-HCl (lysine monochloride) [15], although it may also be produced as liquid L-lysine preparations or in granulated forms. Environmental conditions including temperature, pH, incubation duration, and the choice of carbon and nitrogen sources significantly affect lysine output in bacterial isolates from soil [16]. Because microorganisms require particular cultural conditions and nutrient ranges for maximum productivity, it is necessary to optimize these variables. Consequently, assessing the influence of factors such as temperature, pH, agitation, incubation time, and nutrient sources is vital for enhancing product yield. The objective of this investigation was to optimize the production of free lysine in culture broth using a bacterial strain obtained from diverse soil samples. Specifically, the study focused on identifying microorganisms able to metabolize hydrocarbons and simultaneously synthesize lysine, evaluating the environmental factors that promote growth and enhance lysine accumulation.

## Materials and methods

### Sample collection

Soil samples were collected from different medicinal plants including *Ocimum tenuiflorum, Terminalia arjuna*, *Glycyrrhiza glabra, Abroma augustum, Withania somnifera, Terminalia chebula, Phyllanthus emblica,* *Cassia fistula, Andrographis paniculata, Bombax ceiba, Diospyros blancoi, Azadirachta indica, Justicia adhatoda, Cola acuminate and Terminalia bellirica* of Bangladesh council of scientific and industrial research (BCSIR) Laboratories, Chattogram. Samples were taken in sterile plastic containers from soil at a depth of approximately 5.0 cm and stored at 4 °C.

### Isolation of bacteria

For microbial isolation, the samples were initially processed through serial dilution, after which 0.1 mL of the diluted aliquot was spread onto nutrient agar plates and incubated at 37 °C overnight. Discrete colonies were subsequently subcultured using the streak plate method on fresh nutrient agar media and incubated at 37 °C for an additional 24 hours to obtain pure cultures.

### Primary screening for lysine production

The isolates were evaluated for their ability to produce lysine following the procedure outlined by [17]. Sterile plates of a minimal agar medium composed of KH_2_ PO_4,_ 1.36 g; (NH_4_)_2_SO_4_, 2.0 g; MgSO_4_ ⋅ 7H_2_ O, 0.2 g; CaCl_2_, 0.01g; FeSO_4_ ∙ 7H_2_ O, 0.5 mg; Glucose, 2.0 g; Agar, 12 g; per liter of distilled water were prepared, with the pH adjusted to 7.2 using 1 N NaOH, and autoclaved at 121°C for 15 minutes. The minimal agar plates were then seeded with 24-hour broth cultures of lysine-requiring *Escherichia coli* auxotrophs (DSM 1099 and DSM 5210) and subsequently surface-inoculated with each soil isolate. Following 72 hours of incubation at 37°C, isolates that induced halo formation of the auxotrophic strain were subcultured and selected for subsequent analyses. These isolates, which demonstrated lysine synthesis on solid medium, were classified as lysine producers.

### Production of lysine in a submerged culture

#### Seed inoculum preparation.

A fresh inoculum was generated by transferring a loopful of the bacterial culture into a test tube containing 10 mL of nutrient broth, followed by incubation at 37 °C for 24 hours.

#### Fermentation.

The fermentation basal medium was formulated with KH_2_PO_4,_ 1.0 g; K_2_HPO_4_, 1.0g; MgSO4 ∙ 7H_2_O, 0.4 g; MnSO4 ∙ 7H_2_O, 2.0 mg; FeSO4 ∙ 7H_2_O, 2.0 mg; CaCO3, 50.0 g; dissolved in 1 L of distilled water, and the pH was adjusted to 7.2 using 1 N NaOH. Glucose (20.0 g) and (NH_4_)_2_SO_4_ (10.0 g) were incorporated as the primary carbon and nitrogen sources, respectively. Following preparation, the medium was autoclaved at 121°C for 15 minutes to ensure sterility. Subsequently, 2.0 mL of the prepared seed culture was aseptically transferred into a 50 mL of the sterile fermentation medium and incubated at 37 °C for 72 hours on a rotary shaker operating at 120 rpm to facilitate aeration and uniform mixing.

#### Determination of growth.

The growth of the isolate was assessed turbidmetrically by measuring the optical density of the culture broth at 660 nm using a UV spectrophotometer.

#### L-lysine assay.

The acid ninhydrin technique of Chinard, was used to quantify L-lysine in the supernatant [18]. After centrifugation at 4200 rpm for 30 minutes, the cell-free supernatant was analyzed for L-lysine. In a test tube, 1 mL of glacial acetic acid was mixed with 1 mL of supernatant, followed by 1 mL of a reagent solution containing an acid combination of 0.4 mL of 6 M orthophosphoric acid, 0.6 mL of glacial acetic acid, and 25 mg of ninhydrin per milliliter of the acid mixture. After the test tubes were closed and the reagents thoroughly mixed, they were heated for one hour at 100˚C in a water bath before being cooled. Each tube received 2 mL of glacial acetic acid, resulting in a final volume of 5 mL. A UV Spectrophotometer was used to measure the optical density of the reacting mixture against a blank at 515 nm. Lysine concentration was estimated using a lysine standard curve. The standard lysine curve was created by graphing optical densities versus standard lysine solution concentrations ranging from 0.1 mg/mL to 4.0 mg/mL

### Molecular and phylogenetic identification

Bacterial isolates were inoculated in the NB (nutrient broth) and maintained at 37˚C overnight. A commercial kit from ELK Biotechnology was used to extract the DNA from the bacteria with minimal modifications. For 16S rRNA gene, the universal primers were 27 F (5′-AGA GTT TGA TCC TGG CTC AG-3′) and 1492 R (5′-GGT TAC CTT GTT ACG ACT T-3′) [19]. 25 µL of master mix (Promega, USA), 12 µL of DNA, 2.5 µL of 27 F and 1492 R, and 8.0 µL of nuclease-free water made up the final 50 µL volume for the PCR process. The PCR amplification was carried out following the thermocycling conditions described by Plestenjak with slight modifications [20]. The amplification protocol began with a denaturation step at 95°C for 5 minutes, followed by 35 cycles comprising: denaturation at 95°C for 45 seconds, annealing at 55°C for 1 minute, and extension at 72°C for 2 minutes, with a final extension step at 72°C for 10 minutes. The amplified products were analyzed by electrophoresis on a 1.5% agarose gel, and bands of approximately 1.5kbp were visualized using a gel documentation system, employing a 1 kb DNA ladder (Promega, USA) as a size reference.

Chromatogram viewer and BioEdit were used to modify the chromatogram sequencing files after the amplified outputs were Sanger sequenced. The National Center for Biotechnology Information’s (NCBI) basic local alignment search tool (BLAST) was used to align the collected 16S rRNA sequences with the available 16S rRNA gene sequences submitted in the GenBank database for identification purposes [21]. To determine the taxonomic relationships among the isolates, a phylogenetic tree was constructed in MEGA software (version 11.0) using reference sequences from GenBank. Sequence alignment was performed with the ClustalW method employing the neighbor-joining algorithm. The finalized 16S rRNA sequences of the selected bacterial isolates were then submitted to NCBI GenBank (USA) to obtain accession numbers.

### Optimization of fermentation conditions for lysine production

#### Incubation period.

Lysine production rate was studied from 24 hours to 144 hours, with a 24-hour interval, using the previously mentioned fermentation media.

#### pH.

The impact of different fermentation medium pH values (ranging from 4.0 to 10.0) on lysine synthesis was investigated.

#### Temperature.

The bacterial culture was cultivated at several temperatures of 25˚C, 30˚C, 37˚C, 45˚C, and 50˚C in the fermentation medium in order to optimize the incubation temperature.

#### Carbon sources.

To identify the most suitable carbon substrate for lysine biosynthesis, glucose, lactose, xylose, mannitol, fructose, starch, sucrose, and molasses were individually tested for their effects on lysine accumulation. The carbon source exhibiting superior performance was chosen for additional optimization trials. Subsequently, the influence of increasing concentrations (2%, 4%, 6%, 8% and 10%) of this carbon source on lysine accumulation was examined.

#### Effect of nitrogen sources.

Various nitrogen sources including ammonium sulfate, ammonium chloride, ammonium phosphate, urea, sodium nitrate, yeast extract, beef extract, peptone, casein, and gelatin were evaluated for their impact on lysine production. The most effective nitrogen source identified was subsequently selected for additional experiments. Furthermore, the influence of different concentrations (1%, 2%, 3%, 4%, and 5%) of the selected nitrogen source on lysine synthesis by the organism was assessed.

#### Effect of different growth stimulators.

The effect of 0.1% (w/v) yeast extract, peptone, casein, and their combinations on lysine production was evaluated.

#### Influence of vitamins.

The fermentation process was carried out to evaluate the effect of different concentrations (10.0 µg/mL, 50.0 µg/mL, and 100.0 µg/mL) of vitamins including riboflavin, folic acid, biotin, thiamine HCl, and vitamin C on lysine production.

#### Purification of lysine by ion exchange chromatography.

Purification of lysine was done by ion exchange chromatography according to Robatjazi et al. using a cation exchange resin Amberlite IR 120 [22]. After centrifuging the fermentation broth to produce a clear supernatant, 1N HCl was used to bring the pH of the cell-free culture supernatant to 3.0. After suspending the resin in 1N NaOH, it was repeatedly cleaned with deionized water and then resuspended in 1N HCl. The resin was packed in a (20 x 3 cm) glass column with a stoppered end to adsorb the amino acid. To get rid of all unattached compounds, the column was repeatedly cleaned with water. The compounds of the extract that were bound to the column were eluted by using 1N NaOH. The eluent fractions were collected at a flow rate of 1 mL/min in 5 mL aliquots.

Analytical method: The presence of lysine in purified sample was confirmed by Sykam S 433 automatic amino acid analyzer following the standard protocol.

### Statistical investigation

Three replicas of all the investigations were performed. The data were analyzed using GraphPad prism and Microsoft excel 2016. The mean ± SD values were calculated for each parameter. The results were considered significant when the value of p was < 0.05.

## Result

### Isolation and screening of lysine producers

Soil-derived bacterial communities were first cultivated on nutrient agar to obtain a mixed culture of isolates (Fig 1A). Morphologically distinct colonies were treated as putative species and subsequently sub-cultured on nutrient agar to secure pure isolates (Fig 1B). Each purified soil isolate was then streaked onto minimal agar plates previously seeded with 24-hour broth cultures of lysine-auxotrophic *Escherichia coli* strains (DSM 1099 and DSM 5210). Those isolates that supported halo growth of the auxotrophs, indicating lysine cross-feeding, were purified by sub-culturing and retained for further investigation (Figs 1C and 1D).

**Fig 1:**
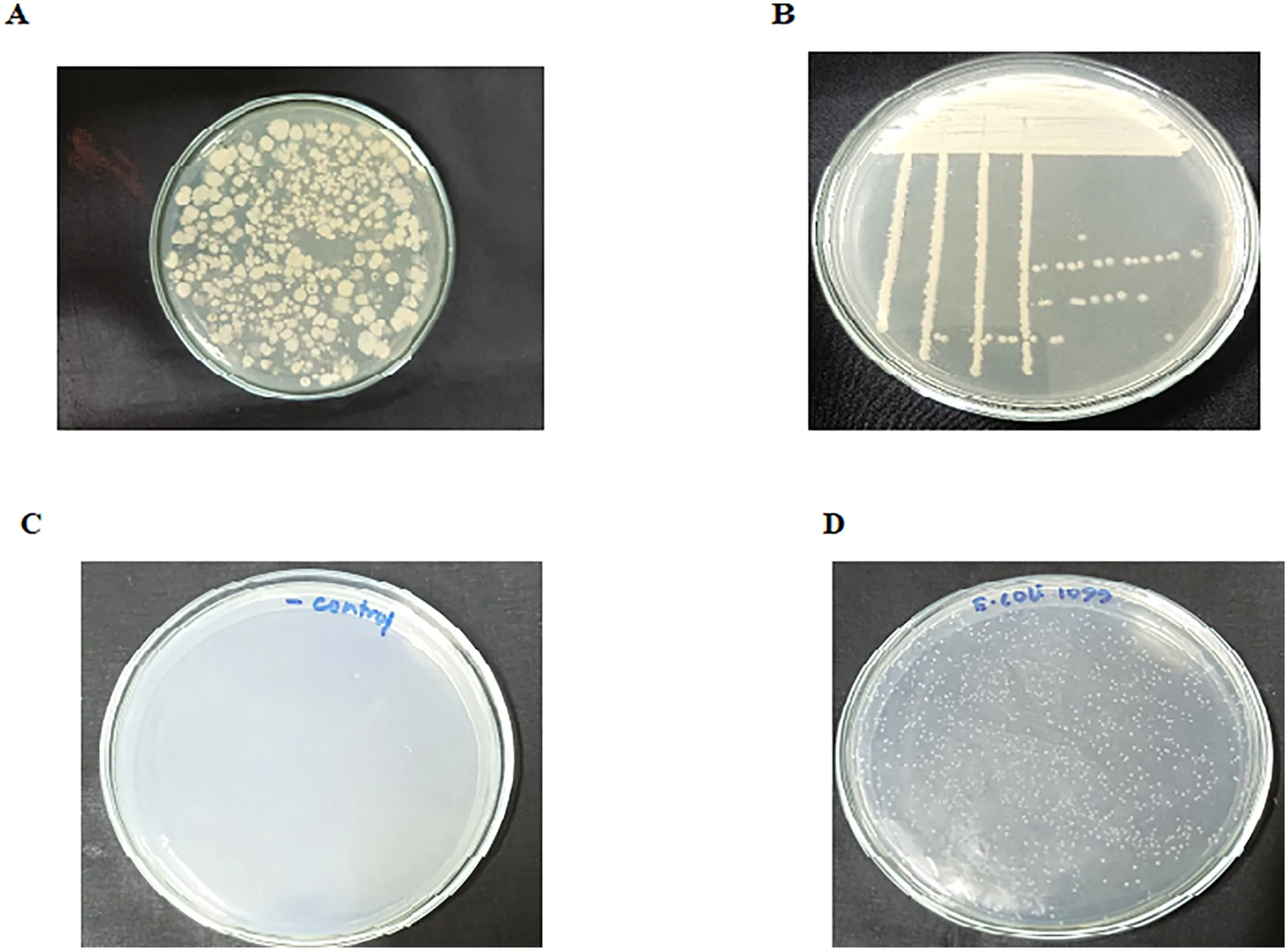
Isolation and primary screening of lysine producing bacteria. A. Isolation of bacteria through serial dilution method, B. Subculture of pure colony, C. Minimal agar media plate spread with only *Escherichia coli* auxotroph (1099) showing no growth, D. Minimal agar media plate spread with *E.coli* auxotroph (1099) and amino acid producing bacteria showing halo growth of *E.coli.*

### Lysine production

The bacterial isolate NTJT4 went through shake flask fermentation method in order to produce crude Lysine. After fermentation, Lysine assay was performed followed by centrifugation. According to the results, highest amount of Lysine (3.49 mg/mL) was produced by NTJT4 isolate (Table 1).

**Table 1:** Production of lysine by different bacterial isolates from soil samples.

Bacterial isolates	Lysine Production (mg/mL)
**NTJT4**	**3.49 ± 0.14**
NTJAM6	3.03 ± 0.21
NTJAM3	3.06 ± 0.18
NTJSO4	2.98 ± 0.08
NTJSH2	2.0 ± 0.14
NTJK3	1.88 ± 0.27
NTJAMB1	2.43 ± 0.25
NTJBS2	3.48 ± 0.41
NTJBS3	3.47 ± 0.23
NTJK15	2.38 ± 0.17
NTJK11	2.81 ± 0.32
NTJK25	2.36 ± 0.29
NTJK21	2.80 ± 0.20
NTJBH24	3.14 ± 0.13
NTJBH12	3.45 ± 0.25

The data are means ± SD from three independent experiments. Here, for NTJT4, NTJ = Lab code, T = Tulsi (*Ocimum tenuiflorum*), 4 = Isolate number; AM = Amloki (*Phyllanthus emblica*); SO=Sonalu (*Cassia fistula*); SH = Shimul (*Bombax ceiba*); K = Kalmegh (*Andrographis paniculata*); B = Basak (*Justicia adhatoda*); BH = Bohera (*Terminalia bellirica*).

### Molecular identification and phylogenetic analysis

Subsequent to the amplification process, the derived PCR products were subjected to electrophoretic separation on a 1.5% agarose gel matrix. The resolved bands were then visualized under ultraviolet (UV) transillumination. The precision of the amplicon’s size was found to be ~ 1.5 kb verified by drawing a comparison with a standard 1 kb DNA molecular weight ladder (Takara), as delineated in Fig 2. Based on its superior lysine-producing capacity, the PCR product of isolate NTJT4 was selected for bidirectional sequencing using forward and reverse primers. The resulting 16S rRNA sequence was analyzed with Chromas and BioEdit, followed by BLASTN comparison against sequences in the National Center for Biotechnology Information (NCBI) database. Further phylogenetic analysis with MEGA software revealed a high degree of similarity to *Bacillus megaterium* (Fig 3) confirming the identity of newly isolated strain NTJT4. The sequence has been deposited in GenBank under the accession number PX498599.

**Fig 2:**
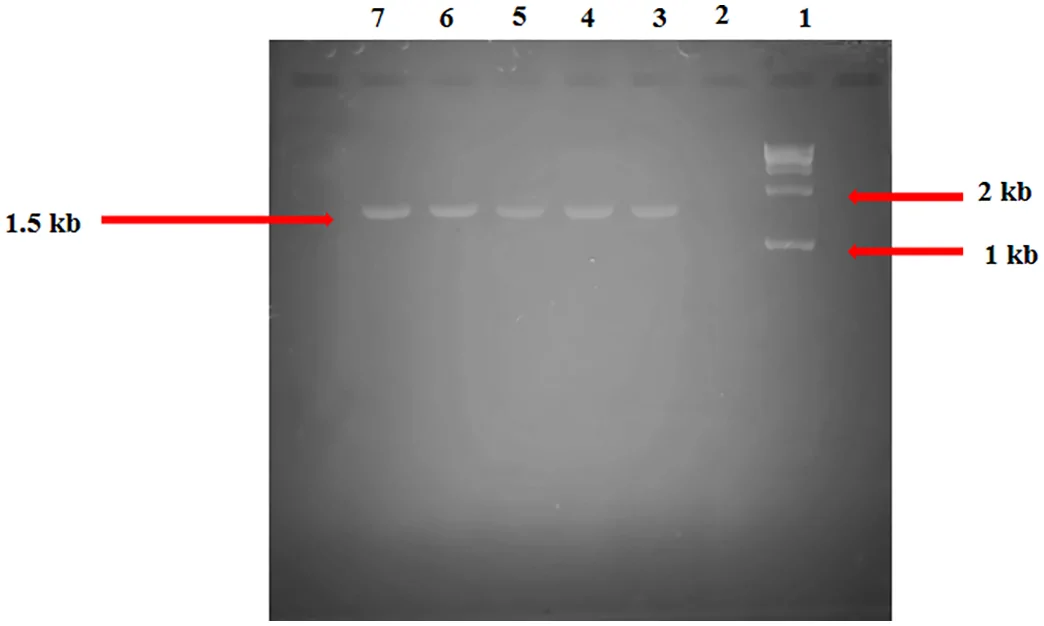
PCR amplification of 16S rRNA gene. Lanes: 1 (1 kb ladder), 2 (negative control), 3 (bacterial isolate NTJT4), 4-7 (other bacterial isolates).

**Fig 3:**
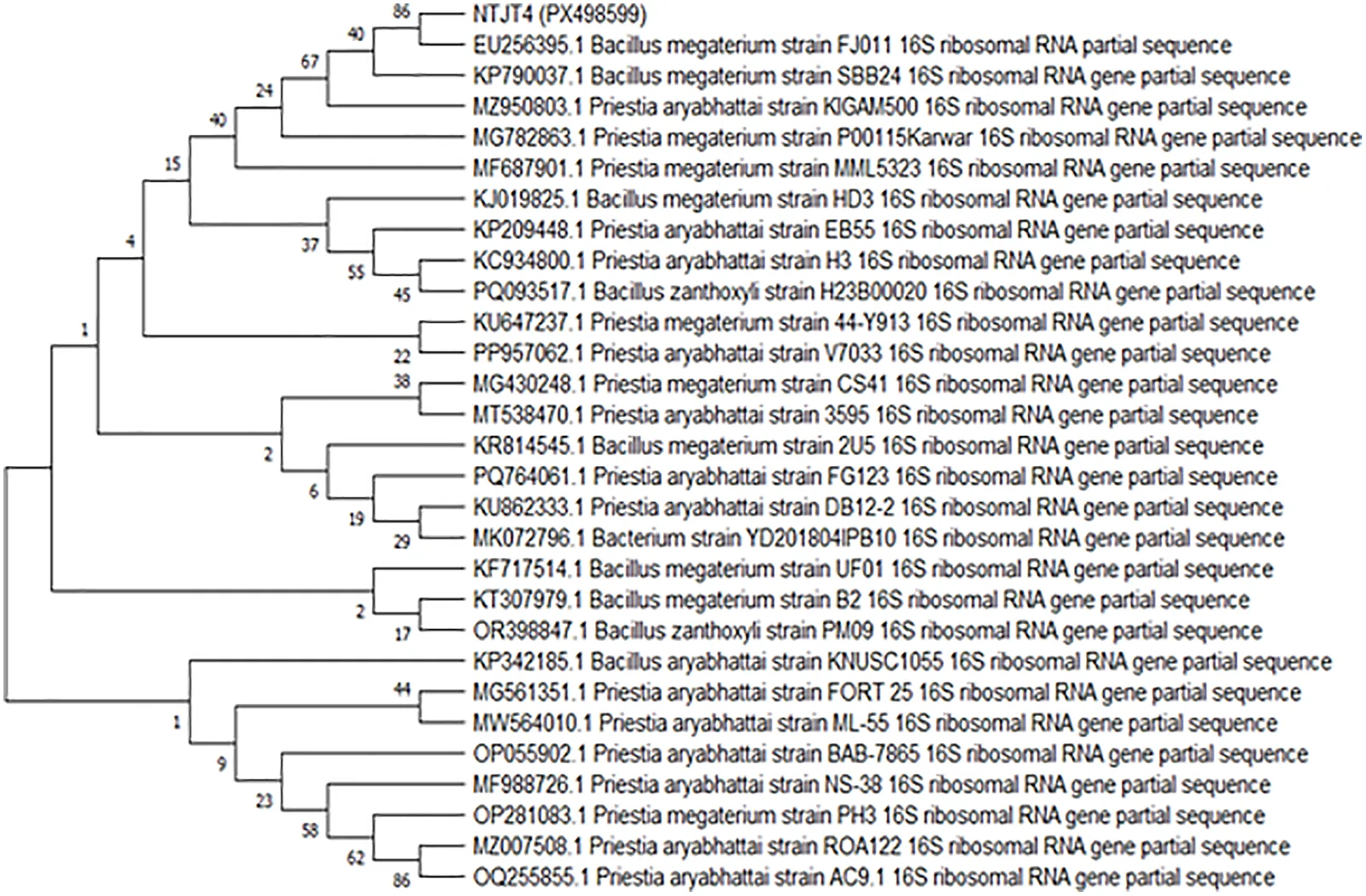
The phylogenetic tree of *Bacillus megaterium* NTJT4 (PX498599).

### Fermentation condition optimization

#### Incubation time.

Optimization of the incubation period for *B. megaterium* NTJT4 showed that there was significant difference in lysine concentration (p < 0.001) and growth (p < 0.001) at different incubation period. Maximum cell growth (OD 2.491 ± 0.06) and lysine production (3.66 ± 0.08 mg/mL) occurred at 72 h (Fig 4). The lowest growth (OD 1.229 ± 0.11) was recorded at 144 h, while the minimum lysine yield (2.67 ± 0.11 mg/mL) was observed at 24 h.

**Fig 4:**
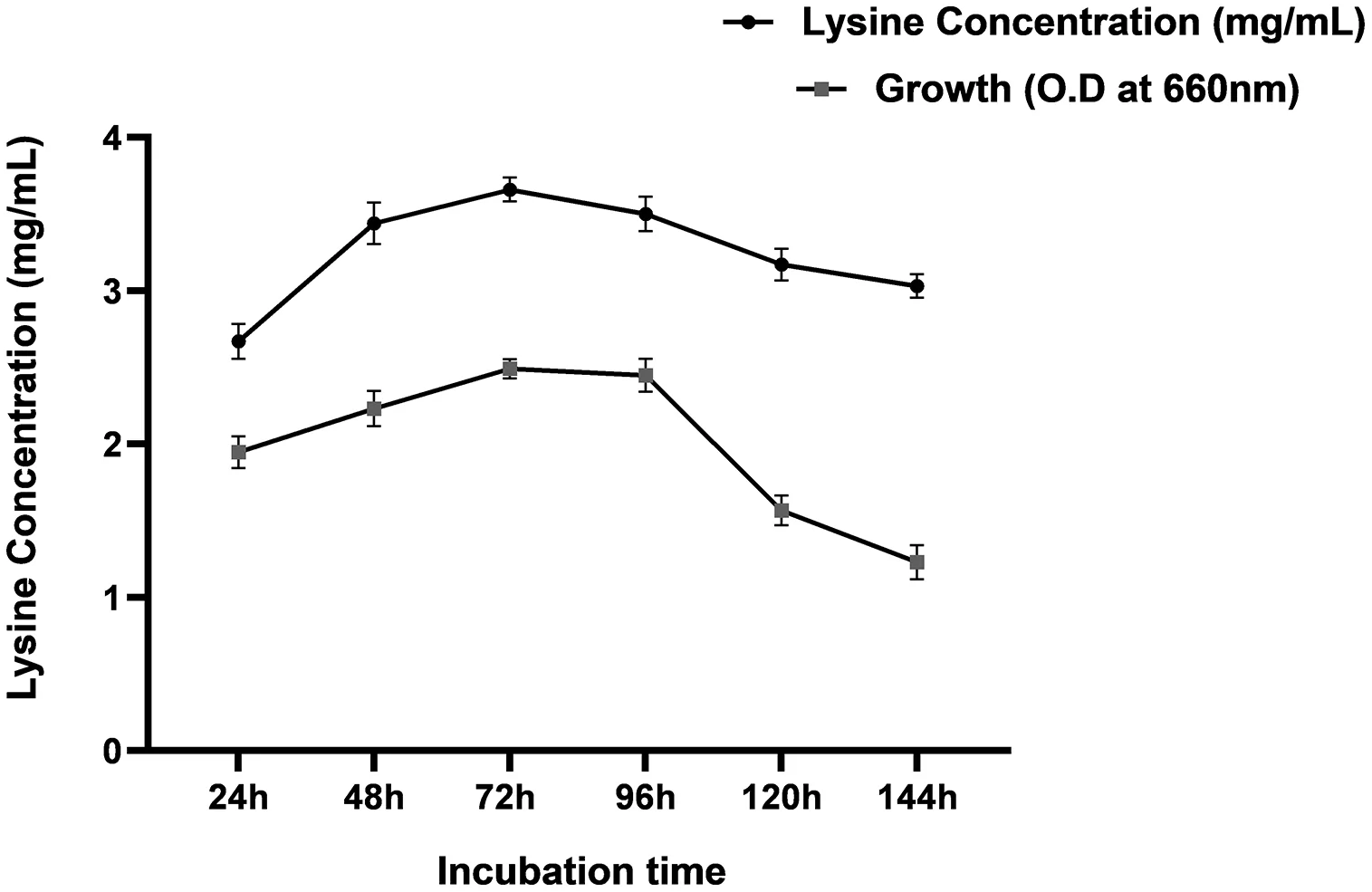
Effect of incubation time on the growth and production of L-lysine *by B. megaterium* NTJT4. The data are means ± SD from three independent experiments.

#### pH.

Varying the initial pH (4.0–10.0) revealed that lysine production and growth significantly differed among pH conditions (p = 0.011 and p < 0.01 respectively). Maximum growth (OD 2.224 ± 0.19) and lysine production (3.67 ± 0.47 mg/mL) occurred at pH 7.0, whereas the lowest growth (OD 1.345 ± 0.22) and lysine yield (1.78 ± 0.80 mg/mL) were observed at pH 10.0 (Fig 5).

**Fig 5:**
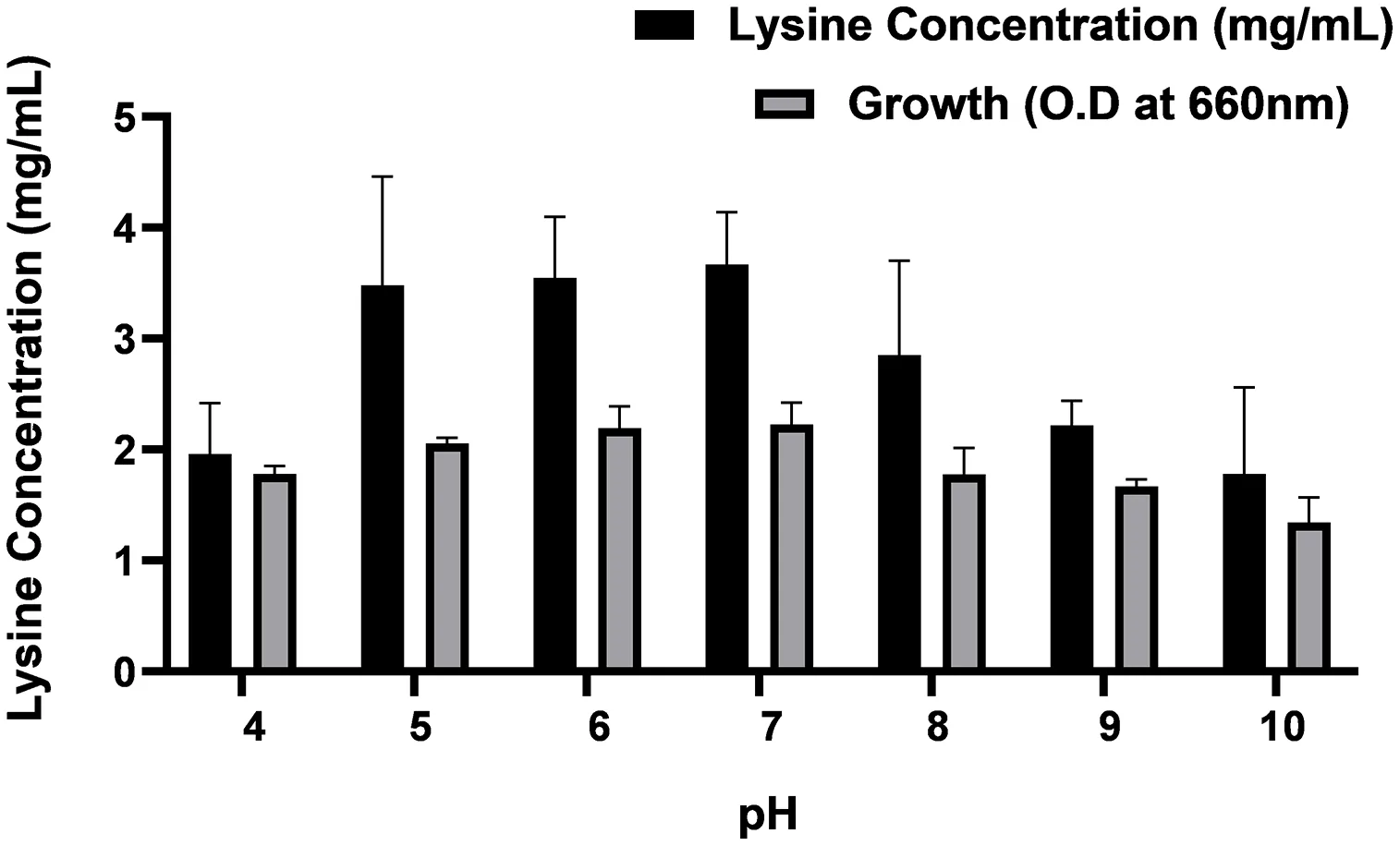
Effect of pH on the growth and production of L-lysine *by B. megaterium* NTJT4. The data are means ± SD from three independent experiments.

#### Temperature.

Temperature optimization showed that both lysine production and growth varied significantly (p < 0.001 both) among different temperatures. Maximum growth (OD 1.381 ± 0.11) and lysine production (4.2 ± 0.18 mg/mL) occurred at 37 °C, while the lowest growth (OD 0.266 ± 0.07) and lysine yield (1.22 ± 0.10 mg/mL) were recorded at 50 °C (Fig 6).

**Fig 6:**
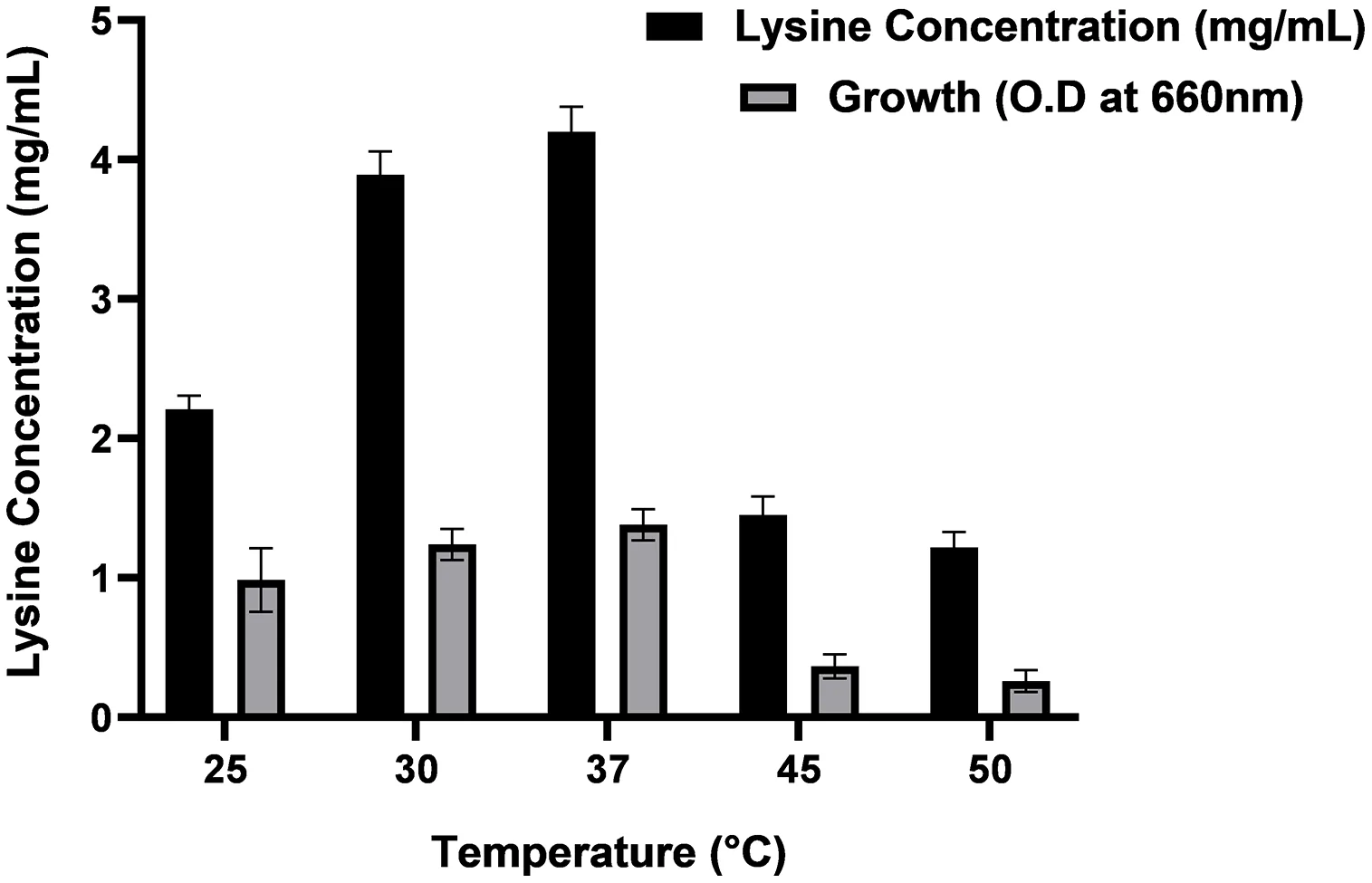
Effect of temperatures on growth and L-lysine production by *B. megaterium* NTJT4. The data are means ± SD from three independent experiments.

#### Carbon sources.

Eight carbon sources (sucrose, glucose, fructose, molasses, starch, lactose, xylose, and mannitol) were evaluated for L-lysine production and showed significantly difference in lysine production and growth (p < 0.001, both). Glucose supported the highest growth (OD 1.215 ± 0.14) and lysine yield (2.49 ± 0.15 mg/mL), while starch resulted in the lowest lysine production (0.178 ± .10 mg/mL) and growth (OD 0.434 ± 0.21) as well (Fig 7). Further optimization of glucose concentration (2–8%) revealed that lysine concentration increased with the increment of glucose concentration but not significantly (p = 0.72, r = 0.22). But lysine production and growth varied significantly among different glucose concentrations (p < 0.001, both). Among these 8% glucose maximized lysine output (4.86 ± 0.20 mg/mL), while 2% glucose resulted in the minimum yield (3.28 ± 0.15 mg/mL) (Fig 8).

**Fig 7:**
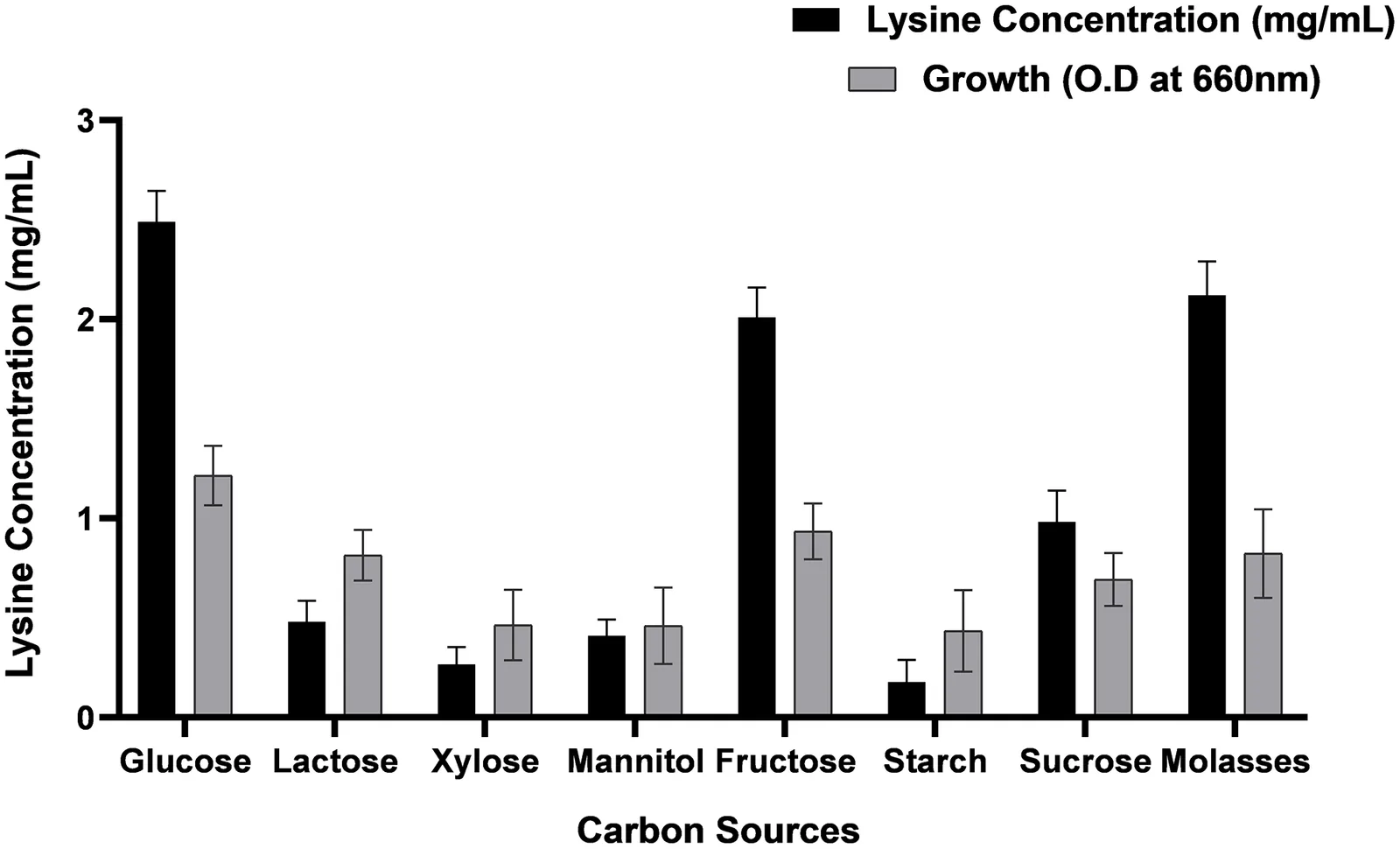
Screening of carbon sources during production of lysine by *B. megaterium* NTJT4. The data are means ± SD from three independent experiments.

**Fig 8:**
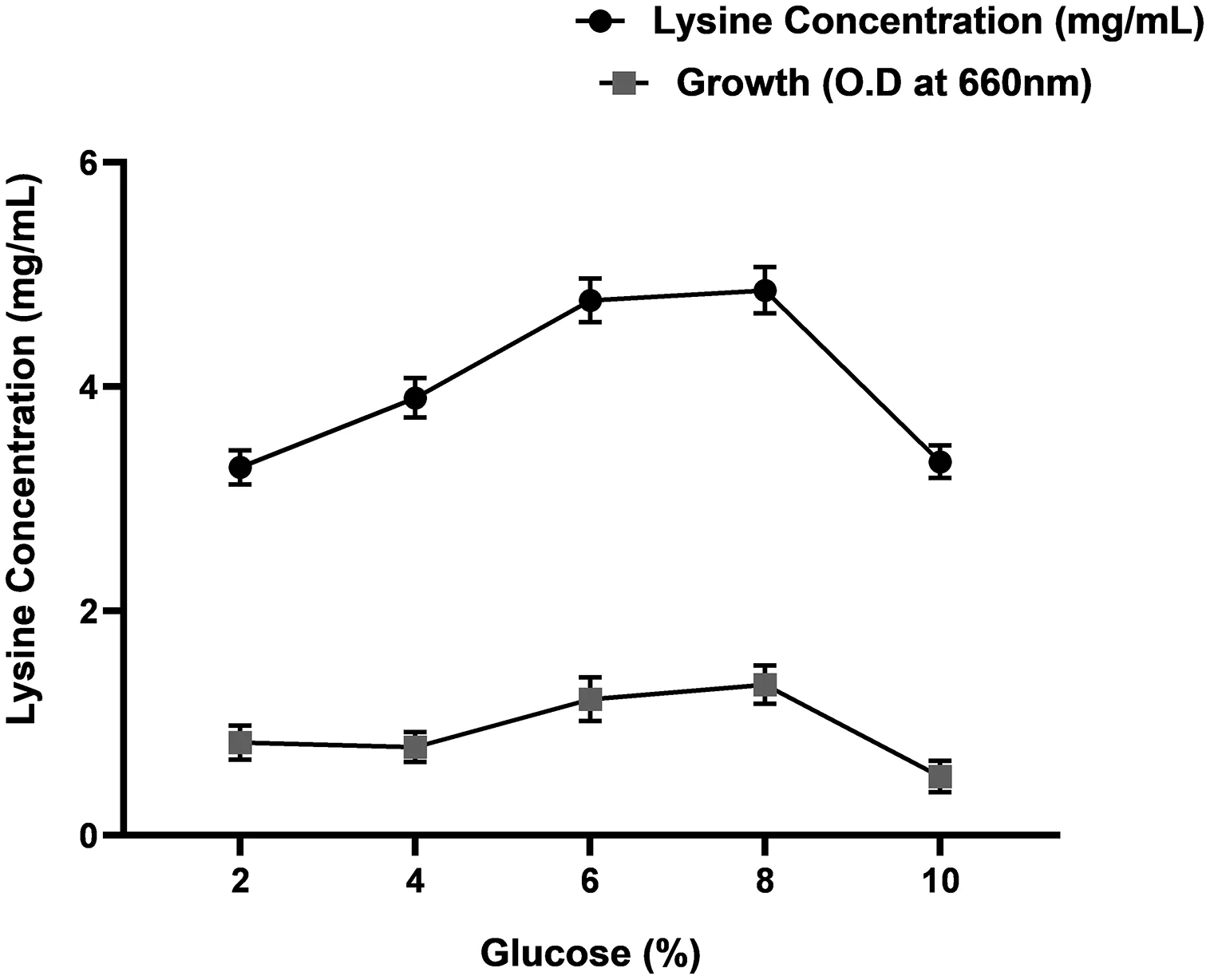
Optimization of glucose concentration in the fermentation medium for biosynthesis of L-lysine by *B. megaterium* NTJT4. The data are means ± SD from three independent experiments.

#### Nitrogen sources.

For the best lysine synthesis, ten distinct nitrogen sources (ammonium sulphate, ammonium chloride, ammonium phosphate, urea, sodium nitrate, yeast extract, beef extract, peptone, caseine, and gelatin) were evaluated in the culture medium. It was found that both lysine production and growth varied significantly among different nitrogen sources (p < 0.001). Maximum growth (OD 2.189 ± 0.28) and lysine yield (3.95 ± 0.14 mg/mL) were observed with ammonium sulfate, while the lowest lysine production (0.089 ± 0.05 mg/mL) occurred with sodium nitrate (Fig 9). Further optimization of ammonium sulfate concentration (1.0–5.0%) showed that lysine concentration and growth significantly decreased with the increment of ammonium sulfate concentration (p = 0.003, r = −0.98 and p = 0.019, r = −0.93 respectively). Lysine production and growth varied significantly among different ammonium sulfate concentrations (p < 0.001 and p = 0.024 respectively). Among these, 1.0% gave the highest growth (OD 0.839 ± 0.19) and lysine production (3.95 ± 0.22 mg/mL), whereas 5% resulted in minimal growth (OD 0.408 ± 0.10) and lysine yield (2.22 ± 0.16 mg/mL) (Fig 10).

**Fig 9:**
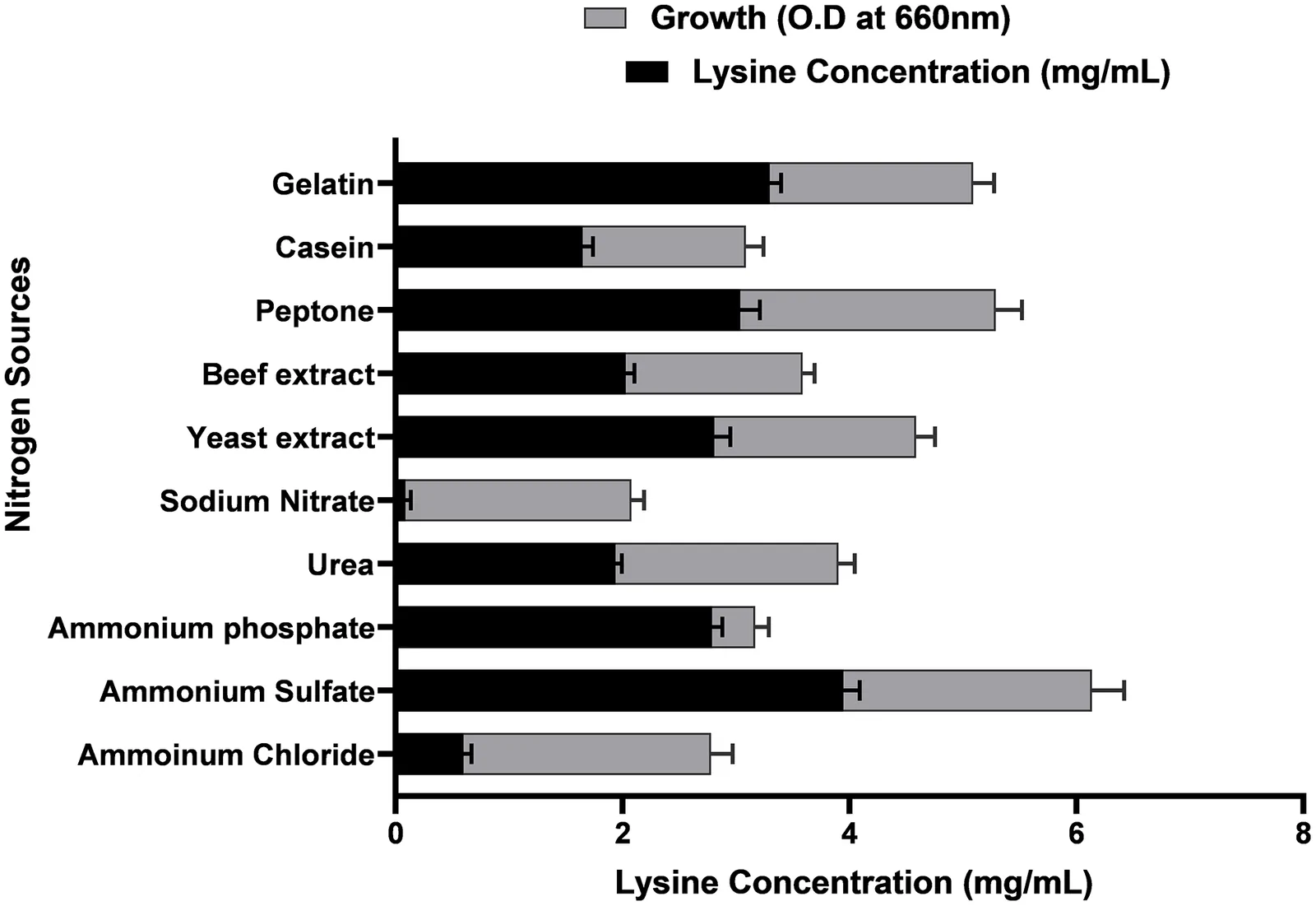
Screening of nitrogen sources for production of L-lysine by *B. megaterium* NTJT4. The data are means ± SD from three independent experiments.

**Fig 10:**
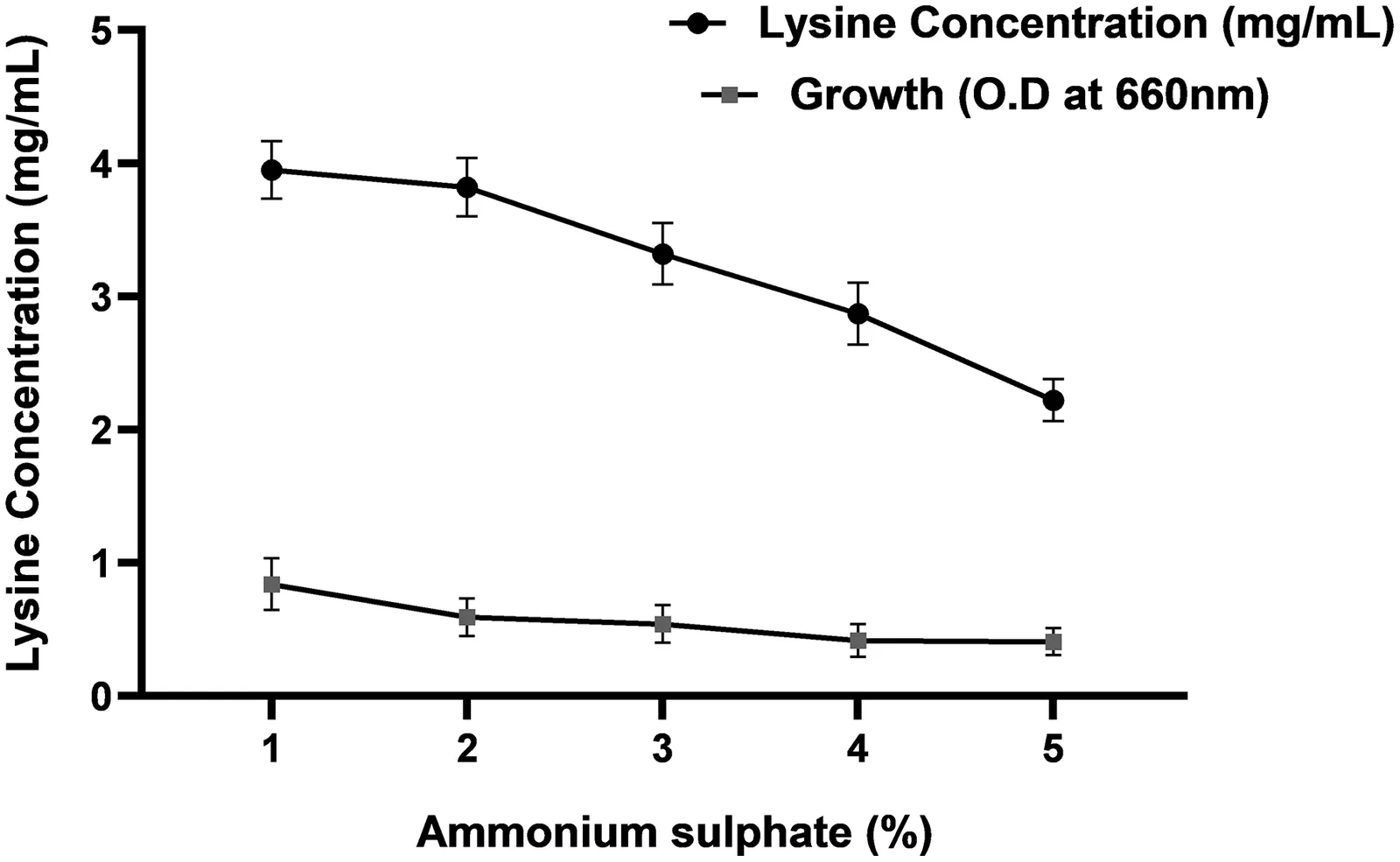
Optimization of amount of ammonium sulphate for production of L-lysine by *B. megaterium* NTJT4. The data are means ± SD from three independent experiments.

#### Growth stimulators.

The effects of different growth stimulators and their combinations on lysine production were evaluated and found that both lysine production and growth were significantly varied among different growth stimulators containing media (p < 0.001 and p = 0.015 repectively. The highest growth (OD 2.043 ± 0.33) and lysine yield (4.86 ± 0.31 mg/mL) were observed in the medium supplemented with a combination of yeast extract and peptone. In contrast, the lowest growth (OD 0.859 ± 0.18) was found in casein+yeast extract combination and lowest lysine production (3.0 ± 0.12 mg/mL) occurred in the medium containing casein (Fig 11).

**Fig 11:**
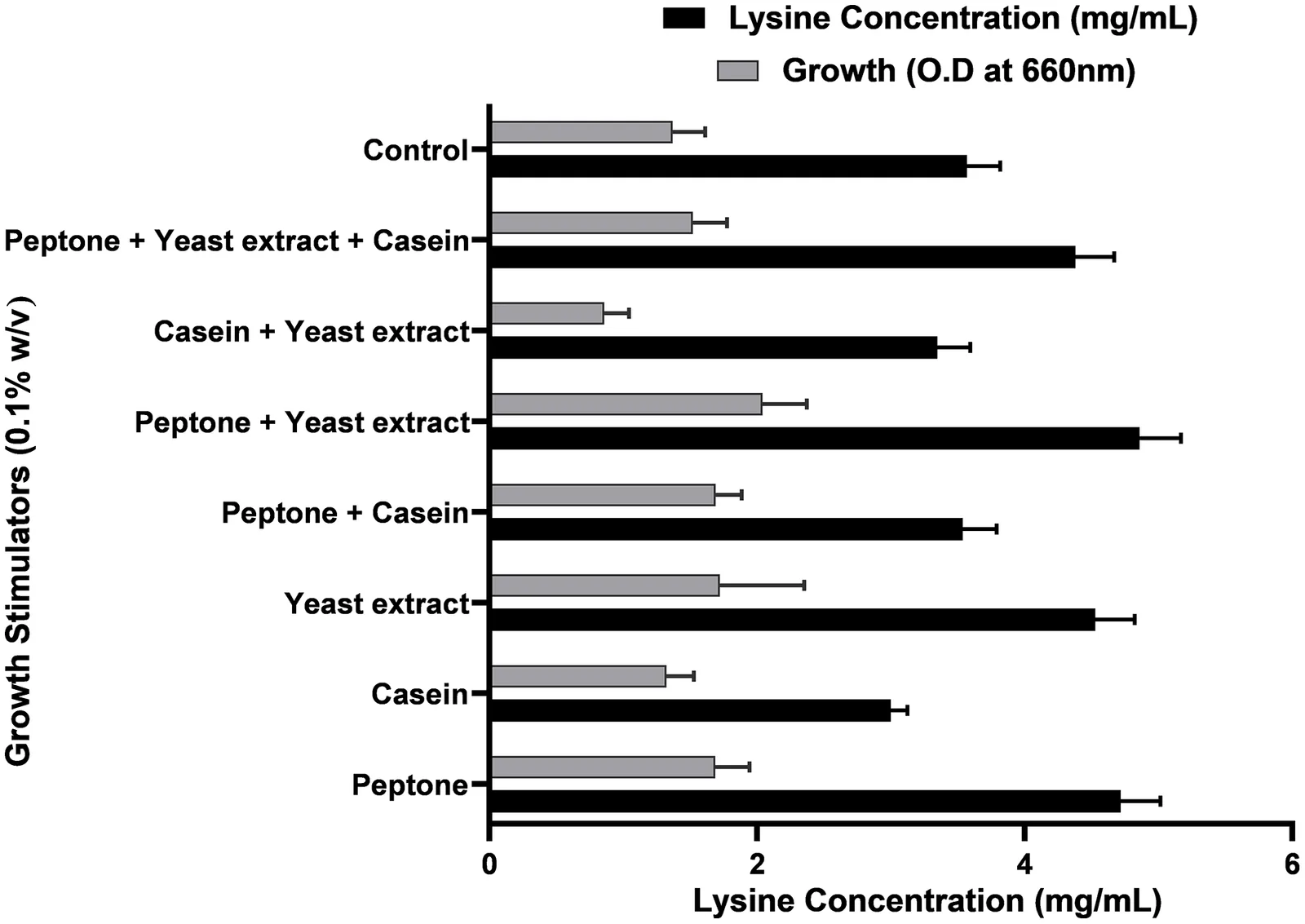
Effect of different growth stimulators on lysine production. The data are means ± SD from three independent experiments.

#### Effects of vitamins at varying concentrations.

The impact of different vitamins (riboflavin, folic acid, biotin, thiamine HCl, and vitamin C) at concentrations of 10 µg/mL, 50 µg/mL, and 100 µg/mL on lysine production was evaluated and results showed that there was significant difference in lysine production (p < 0.001) and growth (p = 0.012) among various vitamin concentrations. The results also indicated that the highest cell growth (OD 2.089 ± 0.31) and lysine production (5.37 ± 0.16 mg/mL) occurred in the medium supplemented with 100 µg/mL of thiamine HCl. In contrast, the lowest growth (OD 1.002 ± 0.19) and lysine yield (2.56 ± 0.12 mg/mL) were observed in the medium containing 10 µg/mL of riboflavin (Fig 12).

**Fig 12:**
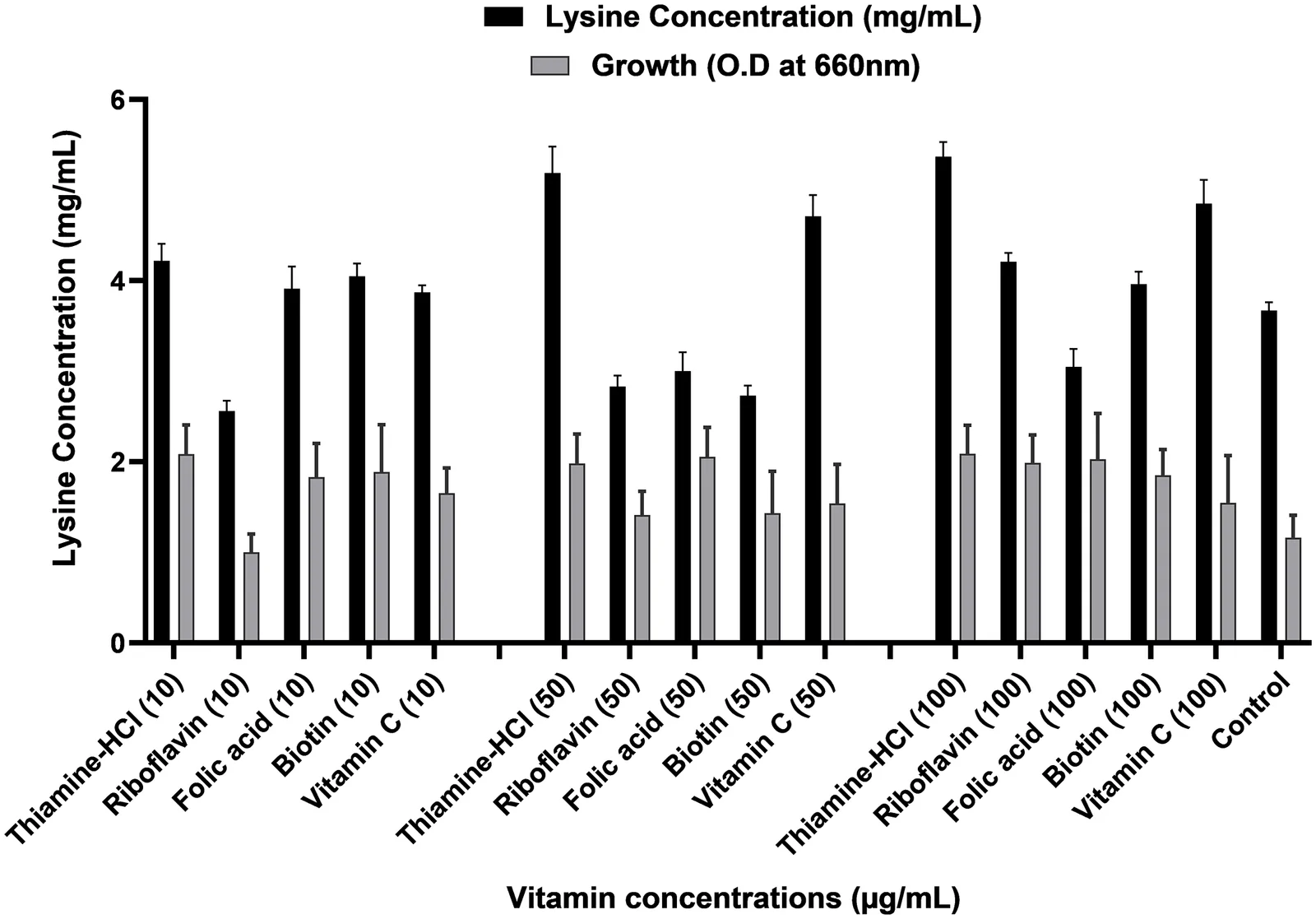
Influence of vitamin concentrations on lysine production. The data are means ± SD from three independent experiments.

### Purification of lysine

Each collected fraction’s lysine content was determined using a quantitative test following purification using ion exchange chromatography on an Amberlite IR-120 ion exchange column. The greatest lysine levels, ranging from 12.78 to 13.44 mg/ml, were found in fractions 13–17. Furthermore, as Fig 13 illustrates, fractions with numbers less than 9 and greater than 23 have either very little or no lysine.

**Fig 13:**
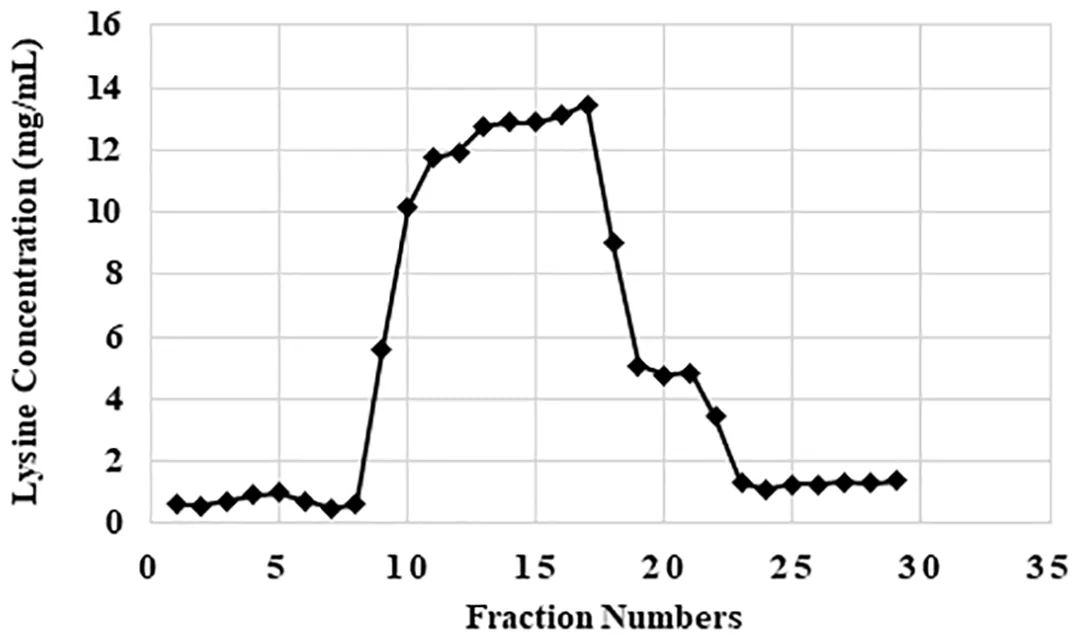
Amberlite IR-120 elution profile of lysine produced by *B. megaterium* NTJT4.

Amino acid analyzer analysis confirmed the presence of lysine in the purified sample (Fig 14). The purified sample exhibited a prominent lysine peak at approximately 39 min, confirming effective enrichment of lysine during purification. This chromatogram is one of the representatives of eluted fractions showing 100% purity of lysine.

**Fig 14:**
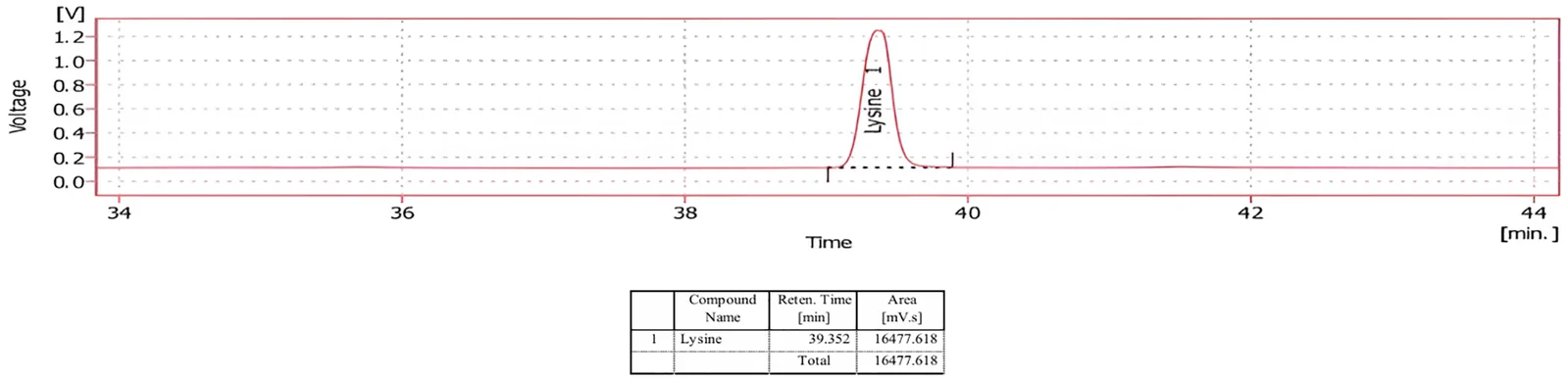
Chromatogram of lysine in purified sample by amino acid analyzer.

The amount of lysine in crude extract at full optimized condition was 6.36 mg/mL which is 1.82 fold higher than the lysine concentration of crude extract before media optimization (3.49 mg/mL) and purification has further increased lysine amount by 2.11 fold from optimized crude extract and 3.85 fold from pre-optimized crude extract (Table 2). The purified lysine showed 100% purity and the recovery yield was 58%.

**Table 2:** Summary of purification of lysine produced by *B. megaterium* NTJT4.

Purification Method	Lysine Concentration (mg/mL)	Purification Fold	Purity (%)	Recovery (%)
Crude Extract^a^	3.49	1.0	–	–
Crude Extract^b^	6.36	1.82	–	100
Purified Product-AIR120	13.44	3.85	100.0	58.0

*a = Before optimization; b= After optimization; AIR-120 = Amberlite IR-120 column chromatography.

## Discussion

Investigating of L-Lysine production by soil isolated bacteria collected from different medicinal plants’ roots contributes to sustainable advancement in the food and feed industries as well as in pharmaceutical industries.

In the present study, the highest amount of lysine produced by soil isolated bacteria *Bacillus megaterium* NTJT4 in its optimized condition was 6.36 mg/mL. The presence of lysine-producing microorganisms in soil is consistent with previous findings. Ekwealor and Obeta recovered a lysine-producing strain*, Bacillus megaterium sp. 14*, from soil, yielding 3.56 mg/mL of lysine in broth culture [10]. Similarly, Sen and Chatterjee reported the isolation of *Arthrobacter globiformis* from soil in Burdwan, India, which accumulated 3.4 g/L of L-lysine in culture [23]. In another study, Sen et al. isolated *Micrococcus* variants from Assam soil, which produced 2.6 g/L of L-lysine in a synthetic medium under optimal conditions [24]. Additionally, *Bacillus* species that produce maximum lysine were isolated from the local soil, according to Umerie et al. [14]. It was also reported that the yielding strains are found mostly among species of *Arthrobacter, Corynebacterium, Brevibacterium* and *Bacillus* [10,25].

After isolation, the primary screening of lysine-producing strains revealed halo growth of auxotrophic colonies, indicating lysine production. This observation is consistent with findings reported in previous studies [26–28].

Sometimes it is difficult to accurately identify different species of bacteria using phenotypic and traditional biochemical characteristics. To distinguish between species and subspecies levels, however, 16S rRNA gene sequencing is essential. Notably, the 5′ region of approximately 275 bp has been identified as a hypervariable (HV) segment with strain-specific signatures, making it particularly valuable for the detection of *Bacillus* species [29]. In the present study, the strain exhibiting the highest lysine production and biomass yield was identified as *Bacillus megaterium* NTJT4 (Accession No. PX498599) based on 16S rRNA gene sequence analysis. Numerous researchers have emphasized the importance of 16S rRNA sequencing for identifying lysine-producing strains [30–32]. The present study was limited to 16S rRNA gene sequencing for strain identification and did not investigate key genes involved in lysine biosynthesis, such as lysC and dapA. Future studies should therefore include PCR-based screening and molecular characterization of key lysine biosynthesis genes to further elucidate the genetic determinants associated with lysine production in the selected strain.

Amino acid producing microorganisms are generally regulated by feedback inhibition, which restricts production under normal physiological conditions [33]. Hence, medium composition and culture conditions are crucial factors that significantly influence fermentation processes and are therefore frequently the focus of rigorous process development and optimization studies. To ensure optimal outcomes, the culture medium must adequately fulfill the nutritional and physiological requirements necessary for efficient microbial growth and product formation. Furthermore, the efficiency of lysine synthesis can be markedly increased by systematically optimizing nutritional components, such as carbon, nitrogen sources, vitamins and growth stimulators, as well as cultural parameters including pH, temperature, and incubation time. The effect of incubation time on lysine production was observed by varying time ranging from 24 h to 144 h and keeping all other variables constant. From the results it was found that *B. megaterium* NTJT4 gave the highest cell growth (OD 2.491 ± 0.06) and lysine production (3.66 ± 0.08 mg/mL) at 72 h. by gradually increasing from 24h culture then started decreasing as the incubation period was increased to 144h. Microorganisms exhibit a specific period of viability during which they achieve peak metabolite production. Once this optimal fermentation window passes, cell viability declines, leading to reduced metabolite yields [34]. For *Bacillus* species, lysine biosynthesis is generally completed within 72–100 hours, a timeframe supported by the findings of Anastassiadis [2]. Adnan et al. through comparative analyses under optimized and non-optimized cultivation conditions, demonstrated that the highest lysine yields typically occur between 48 and 72 hours of incubation, highlighting the sensitivity of production to environmental and nutritional factors [35]. Furthermore, Hussain et al. identified 72 hours as the optimal incubation period for achieving maximal lysine output, reinforcing the notion that fermentation time is a critical parameter requiring precise optimization to enhance product yield [36].

The pH of the culture medium is a critical parameter that significantly affects both microbial growth and metabolite production. In the present study, the highest lysine yield (3.67 ± 0.47 mg/mL) was obtained at pH 7.0. These findings are consistent with the observations of Broer et al. and Rehman et al who reported that an optimal pH range of 7.0–7.5 supports maximal L-lysine production by bacterial isolates [37,38].

The growth rate of microorganisms is strongly influenced by temperature, as it affects the series of biochemical reactions that constitute overall cellular metabolism. In the present study, the effect of temperature on lysine production was evaluated by varying incubation temperatures while keeping all other parameters constant. The increased lysine yields were observed at 37 °C (4.2 ± 0.18 mg/mL) and also at 30 °C (3.89 ± 0.16 mg/mL). Lysine production increased with rising temperature up to 37 °C, beyond which a decline in yield was noted. This reduction beyond the optimal range is likely due to thermal stress, as elevated temperatures can be detrimental to microbial cells, reducing their growth and metabolic activity. Excessive heat also accelerates enzyme kinetics to the point of structural destabilization, ultimately leading to enzyme denaturation and reduced metabolite synthesis. The optimal temperature identified in this study (37°C) is consistent with the findings of Gilani et al. and Mari et al., the latter of whom reported a optimum temperature of 35°C for methionine yield [39,40].

Carbon sources serve both structural and functional roles in microbial cells and are essential for sustaining microbial growth. They exert a significant influence on the synthesis of metabolites and other metabolic by-products. In the present study, glucose supported the highest lysine production (2.49 ± 0.15 mg/mL), demonstrating its effectiveness as a carbon source. This finding is consistent with earlier reports by Nasri et al., Kiefer et al., and Cerning et al., all of whom observed maximal L-lysine yields in media supplemented with glucose [41–43].

Given that lysine biosynthesis peaked in fermentation media containing glucose, it was selected as the optimal carbon source for further optimization. To improve lysine production, glucose concentrations ranging from 2% to 10% were tested. The highest yield (4.86 ± 0.20 mg/mL) was achieved with 8% glucose. These results align with those of Ferreria and Durate who reported that glucose concentrations between 8% and 10% were most effective for maximizing lysine production [44]. The carbon source screening was conducted at a fixed concentration of 2% and so the superior performance of glucose was established only under the tested conditions, and other carbon sources may outperform glucose at different concentrations. Thus, further optimization of alternative carbon sources at different concentrations should be explored in future studies.

An adequate supply of an appropriate nitrogen source is crucial for L-lysine fermentation, as the molecule contains 19.16% nitrogen [44]. In the present study, the highest lysine yield (3.95 ± 0.14 mg/mL) was obtained in medium supplemented with ammonium sulphate. These findings align with those of Ezemba et al. (2016) and Hsiao and Glatz (1996) who also reported enhanced L-lysine production when ammonium sulphate was employed as the nitrogen source [26,45].

Analysis of different concentrations of ammonium sulphate (Fig 10) showed that lysine production peaked at 1.0% (3.95 ± 0.22 mg/mL), after which further increases led to reduced accumulation. This trend is consistent with Ezemba et al. and may be explained by the elevated osmotic pressure associated with high nitrogen levels, which can negatively impact microbial growth and lysine synthesis, as proposed by Pham et al. [26,46]. Similarly, Anakwenze et al. found that a 1% ammonium sulphate concentration resulted in the highest methionine yield by *Bacillus cereus* S8 [30].

In this study, the effects of 0.1% (w/v) growth-promoting substances (peptone, yeast extract, casein, and their combinations) on lysine production were evaluated. As shown in Fig 11 all tested stimulators enhanced lysine accumulation, with the highest yield (4.86 ± 0.31 mg/mL) achieved in the medium containing a peptone + yeast extract mixture. These observations are consistent with earlier reports by Morinaga et al. and Phadatare et al. who demonstrated the stimulatory effects of yeast extract and peptone on metabolite synthesis [47,48]. However, the lowest yield (3.0 ± 0.12 mg/mL) was obtained in the medium supplemented with casein. This reduced response may be due to *Bacillus megaterium* NTJT4’s limited ability to tolerate the casein concentration employed, resulting in diminished stimulatory effects on lysine production.

All the vitamins evaluated in this study enhanced lysine production by *Bacillus megaterium* NTJT4, although their effects varied with concentration. Ekwealor and Obeta (2007) observed that riboflavin stimulated lysine synthesis in *B. megaterium* SP 86 [49]. However, the present study identified thiamine-HCl at 100.0 µg/mL as yielding the highest lysine concentration (5.37 ± 0.16 mg/mL). This might be attributed to the role of thiamine pyrophosphate (TPP), the active form of thiamine, as a cofactor for several enzymes involved in central carbon metabolism, which can influence the availability of precursors and reducing equivalents for lysine biosynthesis [50]. Furthermore, cofactor requirements of enzymes involved in lysine biosynthesis might differ among Bacillus species. Thus the higher lysine yield obtained with thiamine HCl in our strain might reflect greater dependence on TPP-dependent central metabolism and NADPH-generating pathways, whereas the greater response to riboflavin reported in other Bacillus strains may be related to differences in flavoprotein-dependent metabolism.

Ion exchange chromatography is widely used for amino acid purification due to its scalability, simplicity, and high resolution of the eluted molecules from the contaminants [51]. Lysine being a basic amino acid, was adsorbed as a cationic species using amberlite IR120, a strong acid cation resin. The attained lysine purity of 100.0% indicates effective removal of major impurities, while the 58.0% recovery shows that 42% of the initial lysine was retained through the downstream steps due to incomplete elution, and losses during concentration and crystallization steps. For food or pharmaceutical applications, additional chromatographic or crystallization steps would be required to approach increased recovery [52].

## Conclusion

The present study concludes that *Bacillus megaterium* NTJT4 is a highly efficient producer of L-lysine via submerged fermentation. It was observed that the optimum values of fermentation time, pH, temperature, glucose and ammonium sulphate precentage were 72 h, 7.0, 37°C, 8% and 1%, respectively. These findings provide a useful foundation for further development of microbial L-lysine production using this strain. Future studies should focus on improving strain productivity through mutagenesis or metabolic engineering, followed by evaluation of fed-batch fermentation to achieve higher lysine titres and productivity. In addition, scale-up trials in bioreactors are necessary to assess process performance, reproducibility, and economic feasibility under larger-volume conditions. Overall, *B. megaterium* NTJT4 represents a promising candidate for further development toward sustainable and commercially relevant microbial amino acid production and environmental biotechnology.

## Supporting information

S1 Fig Raw gel image of Fig 2 in the manuscript showing PCR amplification of 16S rRNA gene. Here from the right, 1^st^ lane and 8^th^ lane are for 1kb DNA ladder; 2^nd^ lane for negative control; 3^rd^ lane for bacterial isolate NTJT4 and 4^th^ to 7^th^ lane for other bacterial isolates’ 16s rRNA DNA.
(TIFF)
